# Aquaporin expression in the alimentary canal of the honey bee *Apis mellifera* L. (Hymenoptera: Apidae) and functional characterization of Am_Eglp 1

**DOI:** 10.1371/journal.pone.0236724

**Published:** 2020-09-21

**Authors:** Débora Linhares Lino de Souza, Jose Eduardo Serrão, Immo Alex Hansen

**Affiliations:** 1 Departamento de Biologia Geral, Universidade Federal de Viçosa, MG, Brazil; 2 Department of Biology, New Mexico State University, Las Cruces, NM, United States of America; Universidade Federal do Rio de Janeiro, BRAZIL

## Abstract

Aquaporins (AQP) are a family of plasma membrane proteins responsible for water transport through cell membranes. They are differentially expressed in different parts of the alimentary canal of insects where they regulate water transport. These proteins have been studied in detail in some insects, but few data are available for aquaporins of the honey bee, *Apis mellifera*. We used quantitative PCR to study the expression of six putative aquaporin genes in forager honey bees. We found differential expression of all putative AQP genes in crop, midgut, ileum, rectum and Malpighian tubules. We found the entomoglyceroporin *Am_Eglp 1* expressed at extremely high levels in the midgut. We performed a functional characterization of Am_Eglp 1 using heterologous expression in *Xenopus laevis* oocyte followed by water uptake assays. Our results confirmed that the *Am_Eglp 1* gene encodes a functional water transporter. This study shows that all putative honey bee aquaporin genes have complex expression patterns in the digestive and excretory organs of honey bee workers. Our results suggest that Am_Eglp 1 is the principal water transporter in the midgut of *A*. *mellifera* workers.

## Introduction

Aquaporins are plasma membrane proteins facilitating water exchange between cells and their surroundings. They were first discovered in mammalian cells [1–4]. Since then, aquaporins have been found in almost every living organism [5]. These proteins have six conserved hydrophobic transmembrane domains connected by five hydrophilic loops [6]. Sequence analysis revealed high conservation in the transmembrane domains [7, 8]. Two of the loops contain conserved NPA motifs (asparagine, proline, alanine) which form the center of the water channel pore [3]. Water transport can be blocked with mercury (Hg) ions in some types of aquaporins. The presence of a cysteine residue close to the second NPA motif is critical for this Hg sensitivity [8, 9].

A large number of insect aquaporins have been identified, many of them as putative proteins from genome sequencing projects. Insect aquaporins, have been classified in six groups based on their DNA and amino acid sequences and proven or predicted functions: water specific channels (DRIP), water and urea transporters (PRIP), cation channels (BIB), aquaglyceroporins (Glp), entomoglyceroporins (Eglp), and unorthodox aquaporins [10]. In insects, aquaporins were characterized in a variety of species [11–16]. These proteins showed a wide range of functions and expression patterns with important roles in survival and environmental adaptions of insects.

The genome of the honey bee *Apis mellifera* contains six putative aquaporin genes: DRIP (*Am_DRIP*); PRIP (*Am_PRIP*); BIB (*Am_BIB*); Eglp (*Am_Eglp 1*, *Am_Eglp 2* and *Am_Eglp 3*), and one unorthodox aquaporin gene (*Am_Aqp 12L*) [10, 17, 18]. Although individual honey bee aquaporins have been studied in nurse and forager workers [19], expression patterns of all putative aquaporins have not been studied and their functionality remains poorly understood in these bees.

The digestive tract on honey bees is divided in foregut, comprised of crop and proventricular bulb, the midgut, and the hindgut region comprised of ileum and rectum [20]. The crop is critical for nectar transport and storage from the source of food to the hive and responsible for the beginning of honey conversion [21, 22]. Although this organ was considered impermeable because of its cellular features and cuticle lining, water absorption was proven to occur through crop epithelium, contributing to nectar dehydration [23, 24]. The midgut is the main organ of digestion and nutrient absorption, whereas the hindgut and associated Malpighian tubules play important roles in excretion and osmoregulation [25, 26].

In this study we show the relative expression of six putative aquaporin genes in distinct parts of the digestive tract and Malpighian tubules of *A*. *mellifera* workers and present the functional characterization of Am_Eglp 1.

## Results

### Aquaporin expression patterns in the honey bee alimentary canal

**Fig 1:**
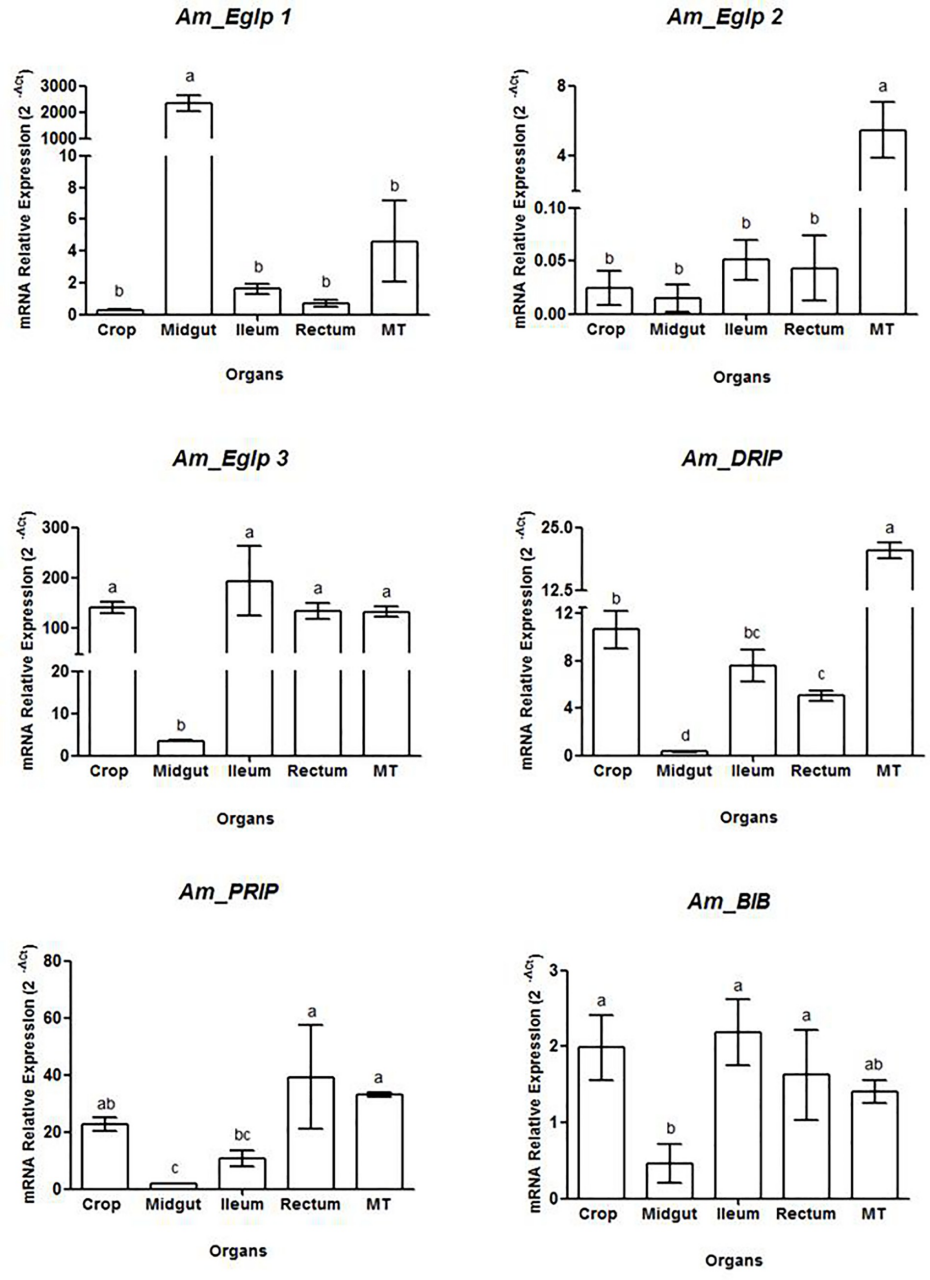
Relative expression of aquaporins genes in the digestive tract and Malpighian tubules of *A*. *mellifera* forager workers. Shown are relative mRNA expression levels determined with qPCR. Values are means ± SD. Means separated by Tukey’s range test (p<0.05). Means which share the same letter are not significantly different. MT: Malpighian tubules.

We performed qPCR analysis for six putative *A*. *mellifera* aquaporin genes. We found that all six were expressed in the digestive tract and Malpighian tubules. *Am_Eglp 1* had higher expression level in the midgut, whereas the other organs showed similar low expression levels (Fig 1A). *Am_Eglp 2* had higher expression levels in the Malpighian tubules (Fig 1B) and *Am_Eglp 3* showed lower expression in the midgut and similar higher expression in the crop, ileum, rectum and Malpighian tubules (Fig 1C). *Am_DRIP* had higher relative expression in Malpighian tubules compared to the other organs (Fig 1D). *Am_PRIP* also had higher relative expression in Malpighian tubules, rectum, and crop with lower expression in ileum and midgut (Fig 1E). *Am_BIB* was lower expressed in the midgut compared to the other organs (Fig 1F).

The midgut is an organ that plays an important role in digestion and nutrient absorption. Because *Am_Eglp 1* expression in the midgut of *A*. *mellifera* was strongly elevated, we chose this aquaporin for additional analysis.

### Bioinformatics analysis of Am_Eglp 1

**Fig 2:**
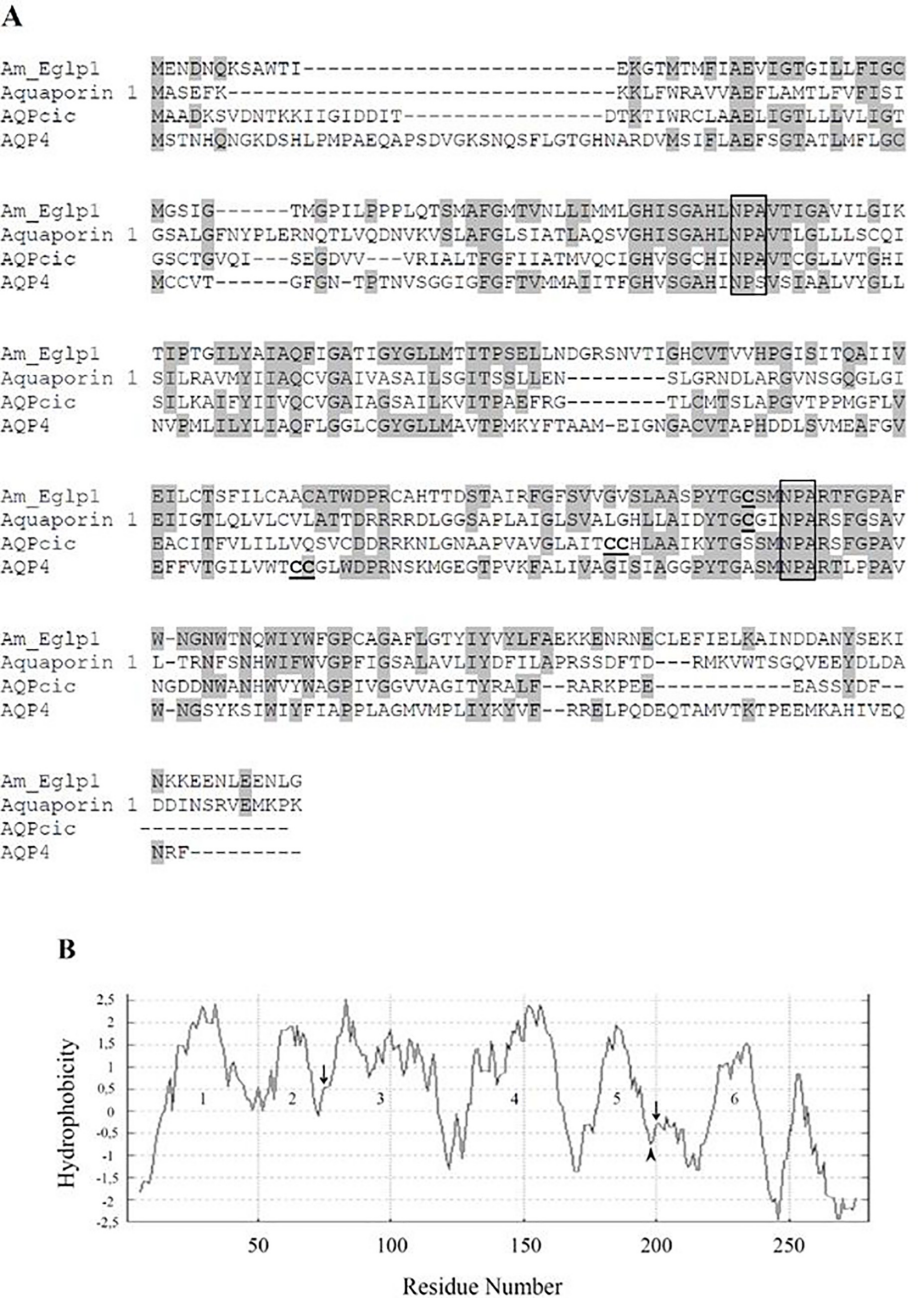
Sequence analyses of Am_Eglp 1. **A**: Amino acid sequence alignment of aquaporins from different organisms with different mercury (HgCl_2_) sensitivity. Am_Eglp 1: *A*. *mellifera* entomoglyceroporin; Aquaporin 1: mammalian aquaporin; AQPcic: *C*. *viridis* aquaporin; AQP4: *A*. *aegypti* aquaporin. Amino acid residues identical to those in Am_Eglp 1 are shaded. NPA motifs are highlighted in a box. **C**: cysteine residues close to second NPA motif. **B**: Hydrophobicity profile of Am_Eglp 1. Numbers 1 through 6 are hydrophobic transmembrane domains. Arrow: NPA motifs. Arrowhead: cysteine position upstream to second NPA motif.

The predicted honey bee protein Am_Eglp 1 has 280 amino acids with a molecular weight of *circa* 30 kDa, according to NCBI online data. Two NPA motifs were found, the first at amino acid positions 76–78 and the second from 200–202 (Fig 2A). There was a cysteine residue on position 197, which is located two amino acids upstream from the second NPA motif.

Amino acids alignments showed that in both Am_Eglp 1 and mammalian Aquaporin 1 a cysteine residue was located at the same distance from the second NPA motif. In AQPcic and AQP4 dual cysteine residues were found 12 and 37 amino acids upstream from the second NPA/NPS motif, respectively (Fig 2A). This result suggests that Am_Eglp 1 is sensitive to inhibition with Hg ions.

The hydrophobicity profile showed that Am_Eglp 1 had six predicted transmembrane domains, hydrophilic N and C termini and five connecting loops (Fig 2B). Loops B and E, located between domains 2–3 and 5–6, respectively, carry NPA motifs (Fig 2B).

### Water uptake assay

**Fig 3:**
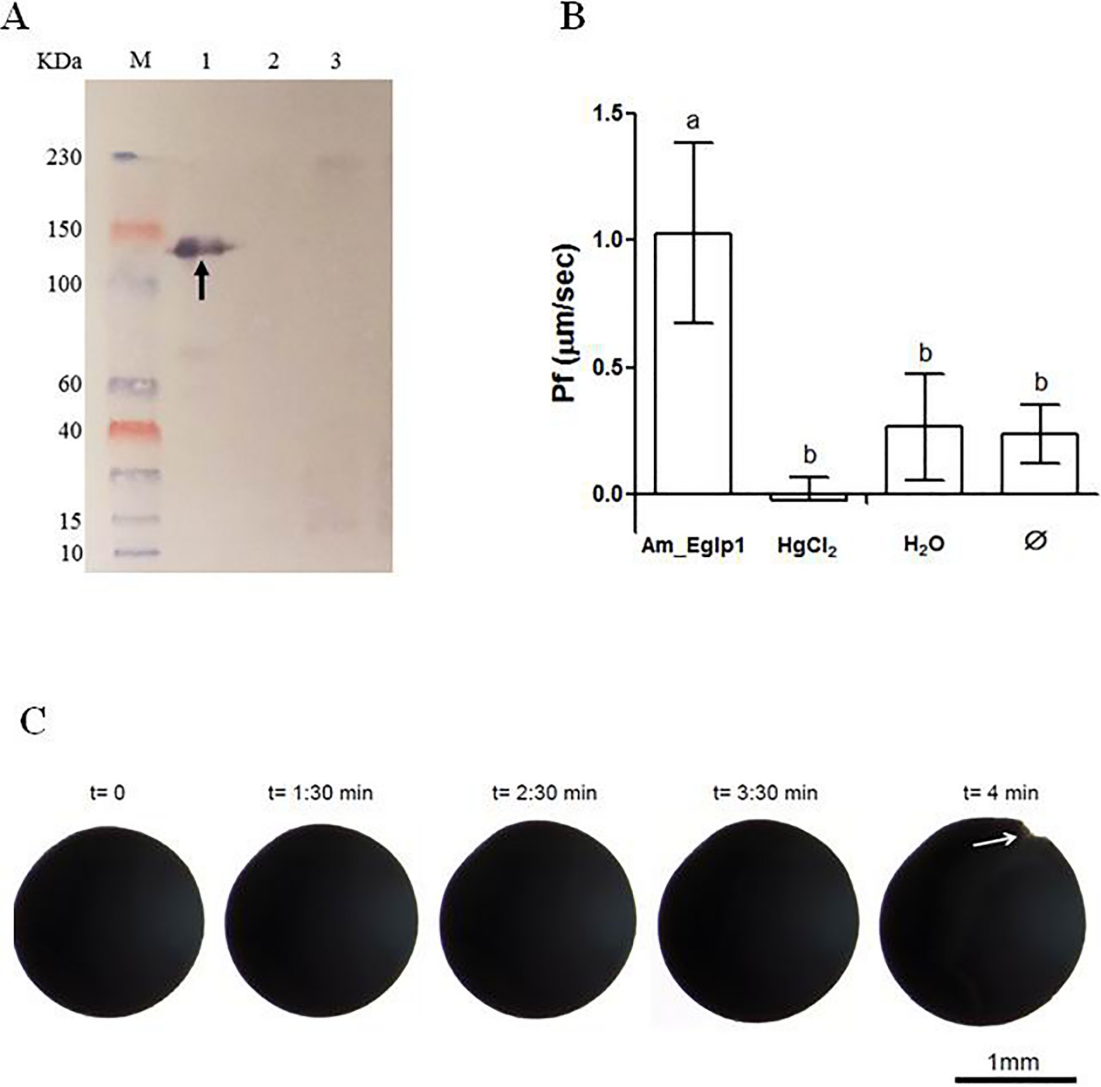
Functional characterization of Am_Eglp 1 via transient expression in *Xenopus* oocytes. **A**: Western Blot analysis of lysed oocytes, using anti myc-tag antibody. M: molecular weight. 1: *Am_Eglp 1* cRNA injected oocytes. 2: water injected oocytes; 3: uninjected oocytes. Arrow: positive reaction of myc-tagged Am_Eglp 1. **B**: Permeability coefficient (Pf) analysis of oocytes expressing Am_Eglp 1 subjected to water uptake assay. Am_Eglp 1: oocytes expressing Am_Eglp 1 (n = 14). HgCl_2_: oocytes expressing Am_Eglp 1 and exposed to mercury prior to water uptake assay (n = 10). H_2_O: water injected oocytes (n = 6). Ø: non-injected oocytes (n = 9). Values are means ± SD. Means separated by Tukey (p<0.05). Means which share the same letter are not significantly different. **C**: Water uptake assay. Oocytes expressing Am_Eglp 1 submitted to hyposmotic schock demonstrated a gradual increase in volume due to water uptake throughout testing time. Arrow: cellular membrane rupture.

cRNA encoding full-length myc-tagged *Am_Eglp 1* was injected into *Xenopus laevis* oocytes. Entomoglyceroporin expression was confirmed by Western Blotting which showed a band with approximately 120 KDa as expected with AQP tetramers with 4 x 30 kDa (Fig 3A). Oocytes expressing Am_Eglp 1 showed higher water permeability compared to both control water injected and non-injected oocytes (Fig 3B). Water uptake was inhibited when oocytes expressing aquaporin were previously placed in HgCl_2_ solution. These oocytes showed the same permeability coefficient as negative controls (Fig 3B). When oocytes expressing Am_Eglp 1 were submitted to a hyposmotic medium they started to gradually swell after a few seconds. Sometimes the water uptake was so high that the cell membrane was not able to endure the cell expansion, resulting in cell membrane rupture and cell burst (Fig 3C).

## Discussion

Aquaporins are well-studied in many insects, from gene structure to protein function [11, 13, 15, 27, 28]. To this day, honey bee aquaporins are only poorly characterized with few studies on gene expression and cellular localization [10, 18, 19, 29]. Considering the importance of honey bees to pollination of wild and cultured plants, ecosystem balance, and economy [30, 31], it is crucial to understand every aspect of these insects’ physiology.

In the mosquito *A*. *aegypti*, six different aquaporin genes are expressed in the alimentary canal and ovary and the expression profile changes with feeding status [13, 16]. In honey bees performing different tasks in the colony, aquaporin genes are also differentially expressed [19]. Forager’s crop and Malpighian tubules showed lower expression of *Am_Eglp 1* compared to nurses’, meanwhile *Am_Eglp 1* is higher expressed in ileum and rectum of foragers, and the same gene is highly expressed in both nurses and foragers’ midgut.

We found that the expression profiles of aquaporins in the digestive tract and Malpighian tubules of *A*. *mellifera* forager workers are organ-specific. As shown in other insects, honey bees express more than one aquaporin in the same cell/tissue [4, 11, 28]. This expression redundancy is a common characteristic in insects, with many types of aquaporins expressed in the plasma membrane and vesicles of different cell types [16, 28, 32]. Redundancy confers advantages in the event of gene inhibition [13, 27]. In *A*. *mellifera*, we observed several aquaporins expressing in different parts of the alimentary canal which suggests that, as in other insects, multiple aquaporins are involved in water transport through cell membranes of this insect.

We confirmed that the entomoglyceroporin *Am_Eglp 1* was highly expressed in the midgut of worker bees, as previously shown [19]. During honey synthesis, water excess needs to be eliminated from the forager’s alimentary canal to avoid dilution of digestive enzymes and osmotic stress [11, 33, 34]. Forager honey bees feed on nectar, which is a diluted food, with up to 90% water [35]. In the midgut of bees, digestive cells have basal plasma membrane invaginations with high numbers of associated mitochondria, indicating cellular absorption activity [29]. The high relative expression level of *Am_Eglp 1* we found in the midgut suggests that this entomoglyceroporin enhances water transport in this organ contributing to digestion and osmoregulation. The other entomoglyceroporins showed a different expression profile, with *Am_Eglp 2* highly expressed in Malpighian tubules and *Am_Eglp 3* highly expressed in other organs but the midgut. These data suggest that *Am_Eglp 2* and *Am_Eglp 3* play a role in osmoregulation, possibly transporting water through the membranes of hindgut and Malpighian tubules cells, as found for aquaporins in different insects [16, 37].

DRIP and PRIP aquaporins are usually found in Malpighian tubules and hindgut of insects [13, 32, 36]. These are the main excretory organs with Malpighian tubules producing the primary urine from hemolymph filtration, whereas ileum and rectum reabsorb important ions and other compounds [26, 37, 38]. Our findings show that *Am_DRIP* and *Am_PRIP* have higher relative expression in Malpighian tubules and hindgut, likely contributing to the high water transport rates in these tissues. DRIP has also been found in honey bee crop cells, where it contributes to nectar dehydration [24]. This data corroborates our findings which show that *Am_DRIP*, *Am_PRIP*, *and Am_Eglp 3* have high expression levels in the honey bee crop suggesting that these three different aquaporins play a role in water transport through crop epithelium.

Aquaporins from the BIB group were first found and described in *Drosophila melanogaster* embryos. BIB expression in *Drosophila* decreases throughout the insect life [39, 40]. Therefore the low expression level of *Am_BIB* we found in *A*. *mellifera* workers may be attributed to their age. BIB aquaporins were previously described as cation channels [41]. Later tests demonstrated that this protein is not related to water transport through membranes, but plays a role in cell—cell adhesion [12]. Therefore, it is reasonable to assume that together with cell junctions, found in digestive tract and Malpighian tubules [29], *Am_BIB* may support tissue integrity in the alimentary canal of *A*. *mellifera*.

Mercury (Hg) blocks certain aquaporin channels via a steric inhibition mechanism. Important for this process are cysteine residues located close to the pores of aquaporins that bind mercury ions non-selectively [42]. For example, the mammalian protein Aquaporin 1 is blocked by Hg due to the presence of a cysteine residue close to the second NPA motif which is located in the extracellular part of the protein [8, 43]. The *A*. *aegypti* AQP4 is an Eglp1 [10], but since it lacks a cysteine residue close to its second NPA motif, this entomoglyceroporin was not blocked by Hg [16]. In *C*. *viridis* the cysteine residue in AQPcic that is likely targeted by Hg is intracellular, thus less accessible. AQPcic was blocked only with high concentrations of Hg [9]. In Am_Eglp 1 a cysteine residue is found in the same position as in mammalian Aquaporin 1. We demonstrated that Am_Eglp 1 was blocked by Hg as well. With this result we concluded that the cysteine residue upstream to the second NPA motif in Am_Eglp 1 is most likely responsible for its Hg sensitivity, because it is accessible for Hg in its extracellular domain. Hydrophobicity predictions showed that Am_Eglp 1 has similar features as some aquaporins functionally characterized [3, 9, 44].

Entomoglyceroporins are known to channel water as well as small neutral solutes such as glycerol and urea. [10, 16, 27, 32]. On the other hand, some entomoglyceroporins, like for example *A*. *aegypti* AQP4, are only permeable for small neutral solutes, and not water [16]. Here we have shown that Am_Eglp 1 is a functional water channel. Further research is necessary to elucidate if Am_Eglp 1 also channels small neutral solutes.

Our results showed that only oocytes expressing Am_Eglp 1 in their membrane increased in volume due to water influx, after being transferred to hypoosmotic solution. Such oocytes did not swell when previously exposed to HgCl_2_ solution, indicating that their water-transport function was blocked by mercury. Together, the data from our functional analysis indicate that Am_Eglp 1 is able to transport water molecules through cell membranes.

### Summary

Our findings show that all six predicted aquaporins are expressed in distinct patterns in the digestive tract and Malpighian tubules of *A*. *mellifera* workers. The entomoglyceroporin *Am_Eglp 1* gene encodes a functional water transporter. Further work is needed to show if this protein also transports other solutes.

## Methods

### Total RNA extraction

Thirty *A*. *mellifera* workers were collected foraging in flowers on the New Mexico State University Campus, Las Cruces. Bees were cryoanesthetized at -20^º^C for 90 s and dissected in PBS saline solution (NaCl 0.1M; Na_2_HPO_4_ 0.1M; KH_2_PO_4_ 0.1M). Malpighian tubules were isolated and the digestive tract divided in crop + proventriculus, midgut, ileum and rectum, yielding three pools of tissue for total RNA extraction. Three biological replicates for each pool were transferred into 500 μL of Trizol^®^ Reagent (Invitrogen). Samples were homogenized with a pellet pestle, incubated for 30 minutes and 100 μL of chloroform was added to each tube, incubated for 10 minutes at room temperature and centrifuged at 12.000 *g* for 10 minutes at 4°C. The supernatant was transferred to 250 μL of isopropanol and incubated at -20°C for 16 hours. Samples were centrifuged again at 12.000 *g* for 10 minutes at 4°C and pellets were washed twice with 70% ethanol, followed by drying and resuspension in 20 μL of nuclease free water. RNA samples were quantified with a Nanodrop 1000 spectrophotometer (Thermo Scientific) and stored at -80°C.

### qRT-PCR analysis of aquaporin transcript abundance

Primer BLAST was used to design specific primers for the six predicted *A*. *melifera* aquaporins and the reference gene *RpL32*—ribosomal protein [45] (Table 1). Reactions were performed using iTaq ™ Universal SYBR® Green One-Step Kit (Bio-Rad), assembled according to the manufacturer's instructions with three independent biological replicates. Each reaction had 50 ng of total RNA, forward and reverse primers at 300 nM in a 10 μL reaction volume. qRT-PCR was performed on Eppendorf Mastercycler ep realplex® (Eppendorf) thermal cycler under the following conditions: reverse transcription at 50°C for 10 minutes, polymerase activation and DNA denaturation at 95°C for one minute, 40 cycles of amplification with denaturation at 95°C for 15 seconds and annealing/extension at 60°C for 60 seconds. Results were evaluated by the 2^-ΔCt^ method [46].

**Table 1:** Data on *A*. *mellifera* aquaporin predicted genes, reference gene, and primers sequence.

Predicted gene	Accession Number (NCBI)	*Primers*	Reference
**Am_Eglp 1**	XM_001121043.4 transcript variant X1	F: CCGCCACCATTACAAACGTC R: ACCGTTGTGCATCCTGGAAT	Finn et al., 2015
**Am_Eglp 2**	XM_006563770 transcript variant X2	F: TGCCCAATGTATCGGTGGAG R: AGGTCGCTAAGAATTCCGCC	NCBI
**Am_Eglp 3**	XM_624191.5 transcript variant X1	F: GCTATCCAAGGCCTCCTTCT R: GACTCGGTGCCAATCAGATT	Finn et al., 2015
**Am_DRIP**	XM_624528.5 transcript variant X2	F: TTGTTTGCCAGTGTTGTGGT R: TCCTCCTTCTGGTTGTCCAC	Finn et al., 2015
**Am_PRIP**	XM_394391.6 transcript variant X2	F: GCAGAATTTCTTGGCACGTT R: CATAGGTGCAATAGCGGGAT	Finn et al., 2015
**Am_BIB**	XM_396705.5	F: GTAGCCGGAGCATCCTCATC R: CAGGGAGGGTCAACAGCAAA	Finn et al., 2015
**RpL32**	XM_006564315.2	F: CCCATAACGTTCTATCTGTGGCA R: CTCGTCATATGTTGCCAACTGG	Lourenço et al., 2008

### Bioinformatics analysis of Am_Eglp1

Potential Am_Eglp 1 mercury (Hg) sensitivity was analyzed according to the distance of cysteine residue to the second NPA motif, as previously described^9^. Am_Eglp 1 amino acid sequence was compared through alignment to other three aquaporin amino acid sequences with well established function and tested Hg sensitivity, using MEGA software version 6.0 [47]. The three sequences used were: aquaporin 1 –mammal—(X70257) [9]; AQPcic—*Cicadella viridis—*(Q23808) [9]; and AQP4—*Aedes aegypti—*(XM_001650118) [16] .50m_Eglp 1 (XP_001121043.2) hydrophobicity profile was assessed with the online software ProtScale^50^, based on Kyte & Doolittle^51^ algorithm, with an 11 residue window.

### Vector construction and cRNA synthesis for heterologous expression in *Xenopus laevis* oocytes

A cDNA containing the complete open reading frame for *Am_Eglp 1* (XM_001121043.4 transcript variant X1) with C-terminal myc-tag was synthesized in vertebrate codon usage by Genewiz (South Plainfield, NJ) and cloned into pXOOM using EcoRI and HindIII restriction sites.

*Escherichia coli* (NEB^®^5-alpha Competent *E*. *coli*, New England BioLabs) were transformed with 50 ng of Am_Eglp1-pXOOM according to manufacturer’s instruction. After transformation, bacterial colonies were selected, cells were transferred to growth medium supplemented with 1:1000 kanamycin, and kept overnight at 37°C in a shaker/incubator at 220 rpm. Plasmid extraction was performed using QIAprep^®^ Spin Miniprep Kit, following the manufacturer’s instruction. DNA was eluted in 40 μL ultrapure water and quantified with a Nanodrop 1000 spectrophotometer (Thermo Scientific). cRNA was synthesized from HindIII-linearized *Am_Eglp 1*-pXOOM vector using the mMessage-mMachine^®^ Kit (Ambion Inc., Carlsbad, CA), with T7 RNA polymerase following the manufacturer’s instruction. Resulting cRNA was quantified and stored at -80°C.

### Oocyte protein expression and Western blotting analyses

De-folliculated *X*. *laevis* oocytes were ordered from Ecocyte Bioscience (Austin, Tx). Each oocyte was injected with 20 ng of cRNA or 30 nL of nuclease free water or kept uninjected. Oocytes were incubated at 16°C for three or four days in modified Barth’s solution (200 mOsm (NaCl 88 mM, KCl 1 mM, CaCl_2_ 0.4 mM, Ca(NO_3_)_2_ 0.33 mM, MgSO_4_ 0.8 mM, Tris-HCl 5 Mm, NaHCO_3_ 2.4 mM, pH 7.3), supplemented with penicillin and streptomycin at 100 mg/ml each.

For Western blot analysis, oocytes membrane was ruptured with tweezers to remove cytoplasm in Barth’s solution. Membranes were lysed in Laemmli Sample Buffer (Bio-Rad), with β-mercaptoethanol 5% and 1μM of protease inhibitor mixture (Sigma-Aldrich), followed by three cycles of heat-shock of 100°C for five minutes and -20°C for 10 minutes. Oocytes membrane protein extracts were resolved on Mini-PROTEAN^®^ TGX™ Precast Gels (Bio-Rad) and electro-transferred to Immune blot-PVDF Membrane for Protein Blotting (Bio-Rad). Membranes were blocked overnight at 4°C in Blocker Blotto in TBS (Thermo Fisher Scientific), followed by incubation with anti-myc-tag antibody (Cell BioLabs) diluted to 1:1000 in blocking buffer for one hour at room temperature. After extensive washes with TBS (50 mM Tris, pH 7.6; 150 mM NaCl), the membrane was incubated for two hours with alkaline phosphatase labeled secondary antibody (Milipore) at room temperature. Bands were visualized with BCIP^®^/NBT Liquid Substrate (Sigma) following manufacturer’s instructions.

### Water uptake assay

Oocytes expressing *Am_Eglp 1*-pXOOM (n = 14), water-injected control oocytes (n = 6), and uninjected oocytes (n = 9) were submitted to hyposmotic shock. Oocytes were transferred from 200 mOsm modified Barth’s solution to 50 mOsm Barth’s solution. Solution was diluted using distilled water. Oocytes were observed for up to four minutes at room temperature while images were obtained every 30 seconds using an Olympus SZX12 stereomicroscope with a Lumen 200 light source and an Ample Scientific TCC3.3 ICE supercooled CCD camera. To test Am_Eglp 1 Hg sensitivity, oocytes (n = 10) were kept for 10 minutes in 200 mOsm Barth’s solution containing 1mM HgCl_2_, prior to water uptake assay. The permeability coefficient (Pf) for each oocyte was calculated using a method previously described [48], with the formula Pf = V_0_ (V/V_0_)/dt S_0_ V_H2O_ (Osm_in_−Osm_out_), where V_0_ = initial oocyte volume, V = final oocyte volume, dt = total time, S_0_ = oocyte surface area, V_H2O_ = water molar volume (18cm^3^/mol), and (Osm_in_−Osm_out_) = Barth’s solution osmolality inside and outside oocytes. (Osm_in_−Osm_out_). Results were used to calculate means and SD prior to statistical analysis.

### Statistical analysis and graph representation

Data from qRT-PCR and water uptake assay were analyzed by one-way variance analysis with post-hoc Tukey at 5% significance. Analyses were performed using R software version 3.1.1 and the packages ‘stats’ and ‘contrast’ [49]. Graphs were designed with GraphPad Prism 5 software.

## Supporting information

S1 Raw images (TIF)
