# Correction: Aquaporin expression in the alimentary canal of the honey bee *Apis mellifera* L. (Hymenoptera: Apidae) and functional characterization of Am_Eglp 1

**DOI:** 10.1371/journal.pone.0248540

**Published:** 2021-03-10

**Authors:** Débora Linhares Lino de Souza, Jose Eduardo Serrão, Immo Alex Hansen

The first author’s initials appear incorrectly in the citation. The correct citation is: de Souza DLL, Serrão JE, Hansen IA (2020) Aquaporin expression in the alimentary canal of the honey bee *Apis mellifera* L. (Hymenoptera: Apidae) and functional characterization of Am_Eglp 1. PLoS ONE 15(9): e0236724. https://doi.org/10.1371/journal.pone.0236724.
