# Supplementary figures and images for: Aquaporin expression in the alimentary canal of the honey bee Apis mellifera L. (Hymenoptera: Apidae) and functional characterization of Am_Eglp 1
Source: PLoS One. 2020 Sep 21;15(9):e0236724. doi: 10.1371/journal.pone.0236724 (PMC7505460; doi:10.1371/journal.pone.0236724)

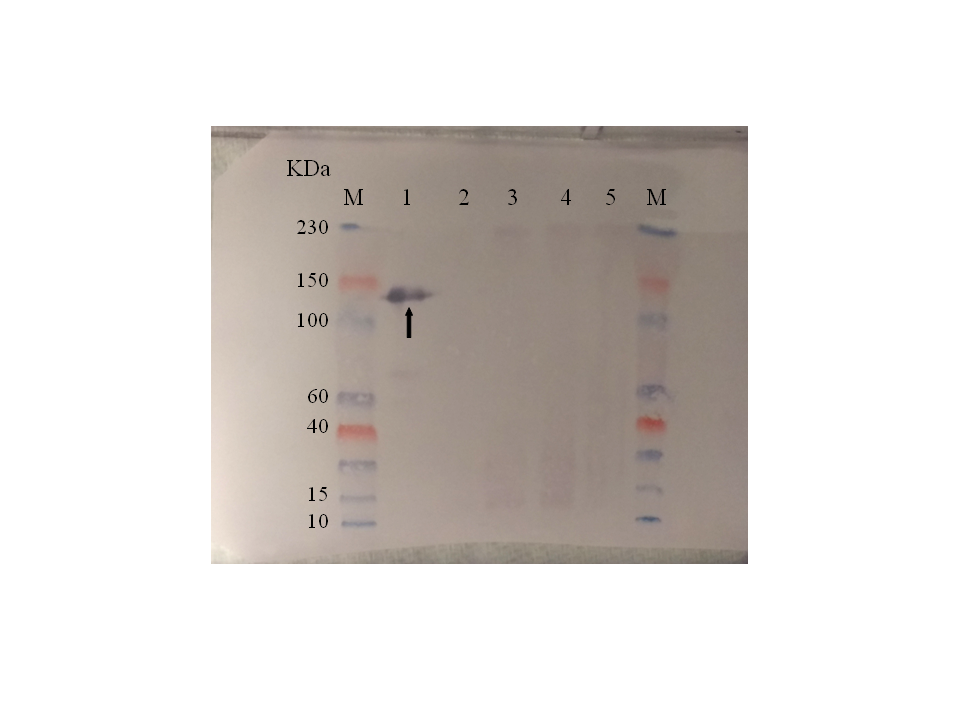

Supplement: S1 Raw images — (TIF) [file pone.0236724.s001.tif]
